# *In vitro *efficacy of a copper iodine complex PPE disinfectant for SARS-CoV-2 inactivation

**DOI:** 10.12688/f1000research.24651.2

**Published:** 2020-10-07

**Authors:** Emily Mantlo, Tanya Rhodes, Jenny Boutros, Laura Patterson-Fortin, Alex Evans, Slobodan Paessler

**Affiliations:** 1 University of Texas Medical Branch at Galveston, Galveston, Texas, USA; 2 Clyra Medical Technologies, Inc., Tampa, Florida, USA; 3 BioLargo Water, Inc., Edmonton, Alberta, Canada; 4 BioLargo, Inc., Westminster, California, USA

**Keywords:** COVID, PPE, iodine, disinfection, decontamination, virus

## Abstract

**Background:** The ability to protect workers and healthcare professionals from infection by SARS-CoV-2, the virus that causes coronavirus disease 2019 (COVID-19), is of great concern. Hospitals, nursing homes and employers are adopting infection control strategies based on guidance from leading public health organizations such as the CDC, OSHA, FDA, and other government bodies. Certain hard surface disinfectants are effective against SARS-CoV-2 but are not suitable for use on skin or personal protective equipment (PPE) that comes into contact with skin. Furthermore, near-ubiquitous alcohol-based hand sanitizers are acceptable for use on skin, but they are not suitable for use on PPE. PPE, especially masks, are also commonly being used for longer durations than normal. There is a need for new products and techniques that can effectively disinfect PPE during wear time without having detrimental effects on surrounding skin. Clyraguard spray is a novel copper iodine complex designed to be used on non-critical PPE.

**Methods:** In this study, the Clyraguard copper iodine complex was tested for its ability to inactivate SARS-CoV-2 in solution.

**Results:** These data indicate the product to be effective in reducing SARS-CoV-2 titers in a time-dependent manner, with the virus being reduced below the detection limits within 30 minutes.

**Conclusions:** These results suggest that Clyraguard may be an effective tool for mitigating cross-contamination of non-critical PPE that may come into contact with SARS-CoV-2.

## Introduction

In late 2019, the world saw the spread of a novel coronavirus (SARS-CoV-2) that caused a global pandemic (ongoing as of this writing) that has claimed the lives of more than 260,000 people and infected 3.8 million as of May 2020 ( Johns Hopkins Coronavirus Resource Center, 2020). The spread of this virus has led governments worldwide to implement unprecedented and far-reaching public health guidelines and lockdowns in an effort to flatten the infection curve and reduce the burden on their respective healthcare systems. Healthcare workers currently have the highest risk of exposure and can easily become vectors of viral transmission. On April 16 ^th^ 2020, the United Kingdom cabinet reported that 16.2% of positive cases were critical workers in NHS ( Centre for Evidence-Based Medicine, 2020), and the United States-based CDC reported that up to 11% of positive cases were healthcare workers in some states ( CDC, 2020a).

Symptomatic COVID-19 patients display symptoms including a dry cough, fever, shortness of breath and sore throat ( CDC, 2020b), and in severe cases can experience severe pneumonia, pulmonary edema, organ failure, and death ( Chen *et al.*, 2020). The virus incubation period varies from 3 to 20 days, during which time a patient can be infectious while asymptomatic, as some evidence suggests ( Bai *et al.*, 2020). While all modes of transmission for SARS-CoV-2 are not completely understood, it is generally believed that the primary mechanism of transmission is through micron-sized droplets and aerosols produced when an infected person, sneezes, coughs, talks or breathes ( WHO, 2020a). Accordingly, public health organizations around the world are urging citizens to practice physical distancing (i.e., “social distancing”), use facemasks when in public, frequently wash their hands, and disinfect commonly touched areas in their surroundings ( CDC, 2020c; WHO, 2020b).

In most countries, many front-line healthcare professionals are required to wear approved N95 masks as well as face shields to protect themselves against infectious droplets and cross-contamination ( CDC, 2020d). However, due to shortages of N95 masks and other protective PPE, health organizations including the CDC are now recommending extended use and reuse of PPE including N95 masks. These new recommended practices may put healthcare workers at additional risk if the PPE cannot be effectively disinfected between uses. Therefore, effective disinfection of protective masks and other PPE before reuse is critical ( CDC, 2020e). One recent study ( Fischer *et al.*, 2020) validated several CDC-recommended PPE decontamination techniques by demonstrating effective disinfection of N95 masks using UV-radiation, dry heat, and hydrogen peroxide vapor application. However, authors showed that while ethanol was effective in disinfecting N95 masks, it also led to reduced filtration capacity in the mask. Another study suggested that ethanol- and chlorine-based disinfectants are not suitable for decontamination due to their capacity to adsorb on fabric material and interfere with their electrostatic properties, thereby reducing filtration capacity ( Liao *et al.*, 2020). In keeping with these findings, bleach, sanitizing wipes, and ethyl oxides are not recommended for use to decontaminate masks ( SAGES, 2020). Recommended PPE decontamination methods (UV, dry heat, hydrogen peroxide vapors) cannot, however, be easily applied during times of extended daily use of PPE, warranting the need for new or additional disinfection methods that can be applied “on the go” in these settings without causing harm to adjacent skin.

Iodine-containing solutions such as povidone iodine (PVP-I) have been proven effective as surface disinfectants against a wide variety of viruses including influenza A, poliovirus, adenovirus type 3, mumps, SARS, MERS, and HIV ( Eggers *et al.*, 2015; Kawana *et al.*, 1997; Wada *et al.*, 2016), with some indication of greater viricidal spectrum of activity compared to other commercially available disinfectants. Furthermore, iodine-based formulations have also been shown to be an effective preventative method to reduce upper respiratory tract infections and oral tract pathogens ( Eggers *et al.*, 2018; Sakai *et al.*, 2008).

Clyraguard copper iodine complex, developed by Clyra Medical Technologies, Inc., is a novel FDA-registered product intended to be used for decontaminating non-critical PPE. The formula has proven antimicrobial activity (FDA submission data, see here) and is cleared for use on skin and wounds. In contrast, other iodine-based products, such as Lugol’s Iodine and PVP-I, may cause staining and skin sensitivity.

In this study, Clyraguard copper iodine complex was assessed for its efficacy in inactivating SARS-CoV-2 using a Vero cell monolayer infection model to provide a basis for whether or not Clyraguard may represent a potentially effective tool to disinfect and prolong the useful protective life of PPE, as well as to provide additional antiviral protection to PPE such as masks.

## Methods

### Cell lines and cell growth

Vero cells were obtained from ATCC and grown in DMEM (Corning) containing 1x L-glutamine and 1% MEM vitamins, and supplemented with 10% FBS (Gibco) and 1% penicillin/streptomycin (Gibco). Cells were incubated at 37°C in 5% CO _2_. Cells were used at 85–95% confluent growth.

### Virus stocks

The SARS-CoV-2 (USA-WA1/2020) virus was obtained from The World Reference Center for Emerging Viruses and Arboviruses (WRCEVA), University of Texas Medical Branch, Galveston, TX. All experiments involving infectious virus were conducted by S.P.’s laboratory at the University of Texas Medical Branch (Galveston, TX) in approved biosafety level 3 laboratories in accordance with institutional health and safety guidelines and federal regulations.

### Preparation of SARS-CoV-2 virus stocks

From the original stock, SARS-CoV-2 was propagated for one passage in infection medium (DMEM containing 1x L-Glutamine and 1% MEM vitamins, supplemented with 1% penicillin/streptomycin (Gibco) and 2% FBS) at 37°C in 5% CO _2_. Briefly, SARS-CoV-2 was added at a MOI of 0.01 to Vero cells and incubated 1 hour at 37°C. Viral inoculum was removed, and fresh infection medium added. Cells were incubated for an additional 48 hours before supernatant was collected. Supernatant was centrifuged for 5 minutes at 3000 rpm to remove cell debris. Virus stocks were stored at -80°C at a concentration of 1x10 ^6^ median tissue culture infectious dose (TCID _50_) per mL.

### Determination of viral titers

Viral titers were measured by TCID _50_ on Vero Cells in 96-well plates. Each log _10_ dilution (10 ^-1^ through 10 ^-6^) of the virus was inoculated in quadruplicates. On day 4 post-infection, the cells were fixed with 10% formalin for 45 minutes and subsequently stained with crystal violet. Cleared wells were quantified to calculate titer.

### Virus inactivation using Clyra or controls

Aliquots of stock virus (10 μl) were mixed with 90 μl of Clyra, diluted Clyra, or control solutions. Clyra was mixed either 1:10 or 1:100 with sterile saline for dilution. Room temperature water was used as a negative control for virus inactivation, while boiling water (water pre-heated to 100°C) was used as a positive control. All mixtures were incubated for 30 seconds, 10 minutes, 30 minutes, or 60 minutes at room temperature, at which time 900 µl infection medium was added to neutralize antiviral activity. Subsequently, the SARS-CoV-2 viral titer (TCID _50_/mL) for each test substance was determined. The experiment was conducted in triplicate.

### Statistical analysis

Statistical significance was determined using a two-way ANOVA with Dunnett’s multiple comparisons test comparing against the control saline group. This was conducted using GraphPad Prism (software version 8.3.0). A p-value of 0.05 or below was considered statistically significant.

## Results

**Figure 1.**
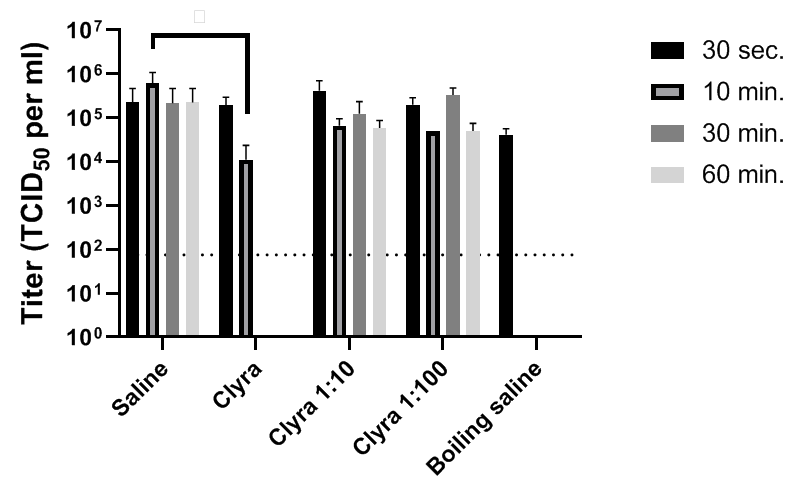
Inactivation of SARS-CoV-2 using Clyraguard. One part SARS-CoV-2 by volume was added to nine parts Clyraguard, diluted Clyraguard, or control saline solutions. Solutions were incubated for the indicated times before titration via TCID _50_.

In this study, the ability of Clyraguard to inactivate SARS-CoV-2 at various timepoints and at various concentrations was assessed ( Figure 1; Table 1). While diluted Clyraguard was unable to inactivate SARS-CoV-2 after any length of time, undiluted Clyraguard was effective at significantly reducing viral titers after just 10 minutes of incubation. After incubation with undiluted Clyraguard for 10 minutes, viral titers dropped by 2 logs (p-value < 0.0001). Furthermore, after incubation with undiluted Clyraguard for either 30 minutes or 60 minutes, viral titers dropped below the limit of detection (<75 TCID _50_ per ml).

**Table 1.** Mean and standard deviation for the SARS-CoV-2 titer (TCID _50_/ml) calculated from three replicates.

	30 sec.		10 min.		30 min.		60 min.	
	Mean	Std. Dev.	Mean	Std. Dev.	Mean	Std. Dev.	Mean	Std. Dev.
**Saline**	2.25 x 10 ^5^	1.95 x 10 ^5^	6.08 x 10 ^5^	3.91 x 10 ^5^	2.17 x 10 ^5^	2.00 x 10 ^5^	2.25 x 10 ^5^	1.95 x 10 ^5^
**Clyraguard**	1.92 x 10 ^5^	8.25 x 10 ^4^	1.12 x 10 ^4^	1.01 x 10 ^4^	<7.5 x 10 ^1^	0	<7.5 x 10 ^1^	0
**Clyraguard 1:10**	4.17 x 10 ^5^	2.36 x 10 ^5^	6.67 x 10 ^4^	2.36E x 10 ^4^	1.25 x 10 ^5^	8.90 x 10 ^4^	5.83 x 10 ^4^	2.36 x 10 ^4^
**Clyraguard 1:100**	2.00 x 10 ^5^	7.07 x 10 ^4^	5.00 x 10 ^4^	0	3.33 x 10 ^5^	1.18 x 10 ^5^	5.00 x 10 ^4^	2.04 x 10 ^4^
**Boiling saline**	4.17 x 10 ^4^	1.18 x 10 ^4^	<7.5 x 10 ^1^	0	<7.5 x 10 ^1^	0	<7.5 x 10 ^1^	0

## Discussion

In this study, Clyraguard copper iodine complex was assessed for its ability to inactivate SARS-CoV-2 in liquid suspension. These data demonstrate that the product has significant viricidal activity against SARS-CoV-2 within 10 minutes and yields complete SARS-CoV-2 deactivation by 30 minutes.

Antiviral activity against human CoV viruses including SARS-CoV and MERS-CoV has been previously been demonstrated using iodine ( Eggers *et al.*, 2015; Eggers *et al.*, 2018). The mechanism of action is not widely understood but researchers have hypothesized that iodine attacks viruses in multiple ways, attacking the protein structure and interfering with hydrogen bonding associated with cysteine, histidine, and tyrosine, thus altering virus membrane structure and causing inhibition of viral release and spread from infected cells ( Qin, 2009).

Iodine-based materials such as PVP-I and Lugol’s solution have shown effective antiviral activity but also exhibit toxicity concerns and can only be tolerated on skin for short periods of time. The copper iodine complex investigated in this study has also been tested according to ISO 10993 standards (FDA submission data, see here) and found to be safe for both skin and wounds, suggesting that it may represent a potential alternative to current iodine-based regimens.

Long-term antimicrobial performance studies with organisms including *Enterococcus faecium*, *Staphylococcus aureus*, *Klebsiella pneumoniae*, *Acinetobacter baumanii*, *Pseudomonas aeruginosa*, *Enterobacter aerogenes*, *Bacillus fragilis* (FDA submission data, see here) have also been conducted with the Clyraguard formula, wherein it was found to be efficacious for up to 72 hours. It is therefore recommended that further studies be carried out under good laboratory practices to directly determine any extended activity against the SARS-CoV-2 virus. This would verify the potential extended use for PPE against SARS-CoV-2, potentially providing additional protection for healthcare professionals working during the COVID-19 pandemic.

In addition, further studies to assess the impacts (or lack thereof) of this copper iodine complex in combination with common face masks such as N95 masks on facial skin and wearer comfort are planned once a suitable and acceptable test method has been established.

Common existing decontamination practices used on PPE such as face masks include UV radiation, dry heat (ovens), and hydrogen peroxide vapor machines such as those approved for use by the FDA manufactured by Battelle and Steris ( Rubio-Romero *et al*., 2020). While substantial evidence exists that supports the consistent antiviral efficacy of hydrogen peroxide vapor PPE decontamination devices ( Steinberg *et al*., 2020), these devices typically require PPE to be reprocessed overnight due to the time required for adequate decontamination. The distinction between these decontamination techniques, which have commonly been used in hospitals during the SARS-CoV-2 pandemic and previous pandemics, and spray-on decontamination products such as the copper iodine complex Clyraguard is that the former require removal of the PPE followed by comparatively lengthy decontamination (typically overnight), whereas the latter is intended to be additional protection on the mask and can be used on-the-go without significant time commitment during a regular work day.

This study demonstrates clear evidence to the potential suitability of the copper iodine complex (Clyraguard) for decontaminating non-critical PPE to help mitigate cross-contamination of SARS-CoV-2. However, this study examined the efficacy of Clyraguard against SARS-CoV-2 only in liquid suspension, and therefore follow-up studies wherein this copper iodine complex is assessed for its efficacy against SARS-CoV-2 (and other viruses) adhered to materials used in personal protective equipment should also be conducted.

## Data availability

### Underlying data

Figshare: *In vitro* efficacy of a copper iodine complex PPE disinfectant for SARS-CoV-2 inactivation - Raw Data in CSV. https://doi.org/10.6084/m9.figshare.12493682.v1 ( Mantlo & Paessler, 2020).

This project contains the raw viral titers for each repeat produced in this experiment.

Data are available under the terms of the Creative Commons Zero "No rights reserved" data waiver (CC0 1.0 Public domain dedication).
