# Supplementary material for: In vitro efficacy of a copper iodine complex PPE disinfectant for SARS-CoV-2 inactivation
Source: F1000Res. 2020 Oct 7;9:674. Originally published 2020 Jul 3. [Version 2] doi: 10.12688/f1000research.24651.2 (PMC7569480; doi:10.12688/f1000research.24651.2)
Supplement: Peer review history

# Peer review: Reviewer response for version 2

**DOI:** 10.5256/f1000research.30025.r72577

**Published:** 2020-10-14

**Authors:** Paul Dabisch

**Affiliations:** 1 National Biodefense Analysis and Countermeasures Center, Operated by BNBI for the US Department of Homeland Security Science and Technology Directorate, Frederick, MD, USA

The authors revisions are satisfactory and I have no additional comments.

Is the work clearly and accurately presented and does it cite the current literature?

Yes

If applicable, is the statistical analysis and its interpretation appropriate?

Partly

Are all the source data underlying the results available to ensure full reproducibility?

Partly

Is the study design appropriate and is the work technically sound?

Yes

Are the conclusions drawn adequately supported by the results?

Yes

Are sufficient details of methods and analysis provided to allow replication by others?

Partly

Reviewer Expertise:

My research focuses on the examination of factors influencing transmission of infectious diseases, including the influence of environmental conditions on the persistence of infectious material on surfaces and in aerosols.

I confirm that I have read this submission and believe that I have an appropriate level of expertise to confirm that it is of an acceptable scientific standard.

# Peer review: Reviewer response for version 1

**DOI:** 10.5256/f1000research.27191.r68324

**Published:** 2020-08-18

**Authors:** Paul Dabisch

**Affiliations:** 1 National Biodefense Analysis and Countermeasures Center, Operated by BNBI for the US Department of Homeland Security Science and Technology Directorate, Frederick, MD, USA

This study describes novel data on the efficacy of a copper iodine complex for inactivation of SARS-CoV-2 in liquid suspension. The results demonstrate that the undiluted copper iodine complex causes a significant decrease in viral infectivity in a cell-based microtitration assay after 10 minutes, with greater than a 5-log reduction in 30 minutes. The methodologies and conclusions are generally appropriate. However, there are several limitations to the study:

1. The study only investigates the ability of the copper iodine complex to inactivate the virus in a liquid suspension. Thus, while the present study provides useful preliminary data, additional testing with virus deposited as droplets or aerosol particles on relevant surfaces needs to be conducted. 
2. As a justification for the use of copper iodine complex, the authors discuss the incompatibility of certain disinfectants for N95 masks, as well as the difficulty of using other decontamination methods while PPE is being worn. However, while the authors cite some evidence that copper iodine complex is safe on skin, the present study does not perform testing to demonstrate the benefit of copper iodine complex in this regard, specifically its compatibility with N95 masks or ability to be used while PPE is being worn. 
3. The manuscript would benefit from additional discussion/comparison of the results obtained in the study to those published for other decontamination methods for SARS-CoV-2, SARS-CoV-1, and MERS.  
4. The methods state that a t-test was used for statistical comparisons. However, it is unclear from the text what comparisons were made. In looking at the data, it appears that either a one- or two-way ANOVA with a multiple comparisons post-test would be more appropriate. 
5. Regarding data presentation, while Table 1 provides mean values, the standard deviation would also be helpful. 

Is the work clearly and accurately presented and does it cite the current literature?

Yes

If applicable, is the statistical analysis and its interpretation appropriate?

Partly

Are all the source data underlying the results available to ensure full reproducibility?

Partly

Is the study design appropriate and is the work technically sound?

Yes

Are the conclusions drawn adequately supported by the results?

Yes

Are sufficient details of methods and analysis provided to allow replication by others?

Partly

Reviewer Expertise:

My research focuses on the examination of factors influencing transmission of infectious diseases, including the influence of environmental conditions on the persistence of infectious material on surfaces and in aerosols.

I confirm that I have read this submission and believe that I have an appropriate level of expertise to confirm that it is of an acceptable scientific standard, however I have significant reservations, as outlined above.

# Response

**Published:** 2020-09-16

**Authors:** Alex Evans

**Affiliations:** BioLargo, Inc., Canada

Thank you for your review. Your comments have provided valuable insights, and we are presently working to edit the paper to address each of the issues raised in your comments. We hope to re-submit this new version in the coming weeks.

# Peer review: Reviewer response for version 1

**DOI:** 10.5256/f1000research.27191.r66377

**Published:** 2020-07-06

**Authors:** Ilya Frolov

**Affiliations:** 1 Department of Microbiology, University of Alabama at Birmingham, Birmingham, AL, USA

This manuscript presents new data, which are important to the field and public health. However, to make the results more reproducible by other groups, the authors need to provide the source of Vero cell and catalog number, number of cells seeded into the wells of 96-well plates and to clarify "infection medium."

Is the work clearly and accurately presented and does it cite the current literature?

Yes

If applicable, is the statistical analysis and its interpretation appropriate?

Yes

Are all the source data underlying the results available to ensure full reproducibility?

Partly

Is the study design appropriate and is the work technically sound?

Yes

Are the conclusions drawn adequately supported by the results?

Yes

Are sufficient details of methods and analysis provided to allow replication by others?

Partly

Reviewer Expertise:

Virology

I confirm that I have read this submission and believe that I have an appropriate level of expertise to confirm that it is of an acceptable scientific standard.
